# Association Between Hemoglobin Glycation Index and Mortality in Critically Ill Patients With Atrial Fibrillation: A Retrospective Cohort Study

**DOI:** 10.1002/clc.70321

**Published:** 2026-04-22

**Authors:** Xianglin Long, Wei Chen

**Affiliations:** ^1^ Department of Dermatology Yueyang Hospital of Traditional Chinese Medicine Yueyang China; ^2^ Department of Cardiology The Third Xiangya Hospital of Central South University Changsha China

**Keywords:** atrial fibrillation, critical care, hemoglobin glycation index, mortality, risk stratification

## Abstract

**Background:**

Effective risk stratification tools are scarce for intensive care unit patients with atrial fibrillation (AF). Although the hemoglobin glycation index (HGI) predicts poor outcomes in various conditions, its clinical utility for critically ill AF populations is undetermined.

**Methods:**

Data from 10,159 ICU patients with AF were extracted from the MIMIC‐IV database. The HGI was calculated as the residual difference between observed and predicted glycated hemoglobin based on fasting glucose levels. Associations with 30‐, 90‐, and 365‐day mortality were assessed using Cox proportional hazards models and restricted cubic splines. Causal mediation analysis was performed to explore underlying pathways.

**Results:**

The lowest HGI quartile (Q1) had significantly higher mortality risks versus Q2 (reference) across 30‐day (HR = 1.46), 90‐day (HR = 1.42), and 365‐day (HR = 1.26) follow‐ups. A non‐linear, inverse association was confirmed, with risk concentrated at low HGI levels. This association was stronger in patients with elevated BMI (*p*‐interaction < 0.01). Causal mediation analysis revealed that red blood cell distribution width (RDW) significantly mediated the risk of low HGI, explaining 3%–8% of the total effect.

**Conclusions:**

The study demonstrates a non‐linear inverse association between HGI and mortality in critically ill AF patients, a relationship partially mediated by RDW and particularly strong in patients with elevated BMI. The findings highlight that the increased risk is primarily concentrated among patients with the lowest HGI values.

## Graphical abstract

In 10,159 critically ill patients with atrial fibrillation, a low hemoglobin glycation index (HGI) independently predicted higher short‐ and long‐term mortality. This association was significantly stronger in patients with elevated BMI and was partially mediated by red blood cell distribution width (RDW), supporting HGI as a potential risk‐stratification biomarker.

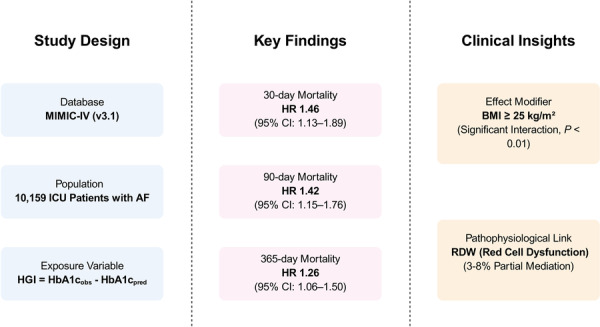

## 1 Introduction

Globally, atrial fibrillation (AF) impacts more than 33 million people and substantially increases the likelihood of stroke, heart failure, and mortality [1]. In intensive care units (ICUs), critically ill patients with AF are at heightened risk due to multiorgan dysfunction and metabolic imbalances, yet there is a shortage of well‐developed prognostic tools for this group [2, 3].

Glycemic dysregulation, commonly assessed using hemoglobin A1c (HbA1c), predicts cardiovascular outcomes but does not account for interindividual variations in the relationship between HbA1c and mean glucose levels [4]. The hemoglobin glycation index (HGI), derived as the residual from a regression of HbA1c on fasting glucose, quantifies intrinsic glycation propensity independent of acute glucose fluctuations [5, 6]. Proposed by Hempe et al., HGI captures individual variations in hemoglobin glycation rate, a key determinant of HbA1c beyond blood glucose levels [7, 8]. Given the metabolic and inflammatory perturbations common in critically ill patients, HGI may offer unique insights into their glycemic and inflammatory status.

Although HGI has been validated as a prognostic marker in diabetes and coronary artery disease, its relevance in critical ill AF remains unknown [9, 10]. This gap is significant given the high prevalence of metabolic comorbidities in AF patients and HGI's ability to reflect inflammatory and metabolic risks beyond conventional glycemic metrics [11]. Furthermore, emerging evidence of non‐linear relationships between HGI and mortality in cardiovascular diseases highlights the need for more nuanced approaches to risk stratification [9, 12].

Using data from the MIMIC‐IV database, this study aimed to investigate the association between the HGI and all‐cause mortality at 30, 90, and 365 days in critically ill patients with atrial fibrillation. In addition, restricted cubic spline models were employed to explore potential non‐linear relationships and subgroup analyses were conducted based on age, BMI, and comorbidities to identify populations at elevated risk.

## 2 Materials and Methods

### 2.1 Data Source

This retrospective cohort study conducted an analysis of de‐identified clinical data sourced from the Medical Information Mart for Intensive Care IV (MIMIC‐IV v3.1), a publicly accessible database containing records of over 65,000 patients admitted to intensive care units at Beth Israel Deaconess Medical Center in Boston, Massachusetts, spanning the years 2008 to 2022 [13, 14]. The establishment of the MIMIC‐IV database was approved by the Institutional Review Boards of the Beth Israel Deaconess Medical Center (Approval No. 2001‐P‐001699/14) and the Massachusetts Institute of Technology (Approval No. 0403000206). Because the dataset comprises fully de‐identified health information, the requirement for individual patient consent for this retrospective analysis was formally waived. The research adhered to the STROBE guidelines and the ethical principles outlined in the Declaration of Helsinki. The author Xianglin Long successfully completed the online training course on the protection of human research participants offered by the U.S. National Institutes of Health (NIH). Upon receiving certification (Certificate No. 68,822,776), access to the dataset was granted.

### 2.2 Study Population

**Figure 1:**
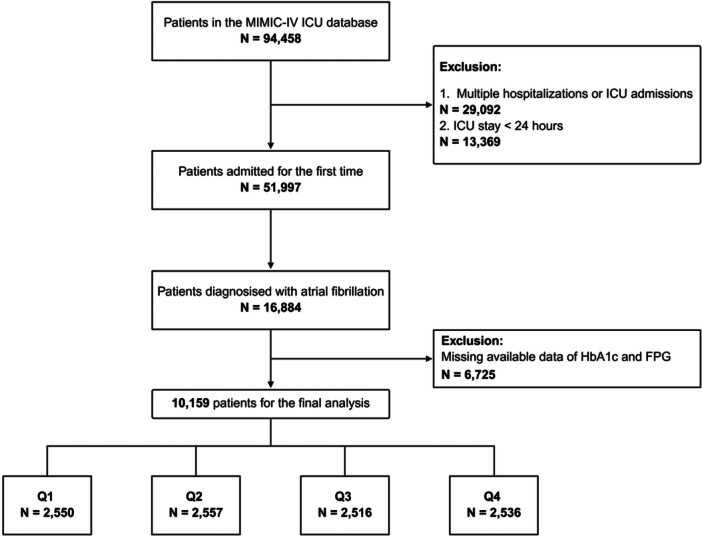
Flowchart of the study cohort.

Adult ICU patients diagnosed with atrial fibrillation were identified through ICD‐9/10 diagnostic codes, as detailed in Table S1. The exclusion criteria included patients with multiple hospitalizations, missing baseline glycemic markers (fasting blood glucose (FPG), and glycosylated hemoglobin HbA1c) within the first 24 h of ICU admission, and those with ICU stays of less than 24 h. This selection process was designed to minimize survivorship bias and to ensure a rigorous evaluation of glycemic parameters. The patient selection process and study flow are depicted in Figure 1.

### 2.3 Demographical and Laboratory Variables

Baseline characteristics and laboratory data were extracted utilizing Structured Query Language (SQL) with standardized ICD‐9/10 codes. We gathered demographic variables, including age, sex, and body mass index (BMI), as well as comorbidities such as hypertension, diabetes, myocardial infarction, congestive heart failure, chronic pulmonary disease, renal disease, and malignancy. Admission laboratory values collected comprised white blood cell (WBC) count, red cell distribution width (RDW), hemoglobin, platelet count, international normalized ratio (INR), creatinine, HbA1c, and FPG. Additionally, data on drug treatments, including insulin, vasoactive drugs, and β‐blockers, were collected. Severity‐of‐illness scores, specifically the Sequential Organ Failure Assessment (SOFA) and Acute Physiology Score III (APS III), along with insulin administration data, were also gathered.

**Figure 2:**
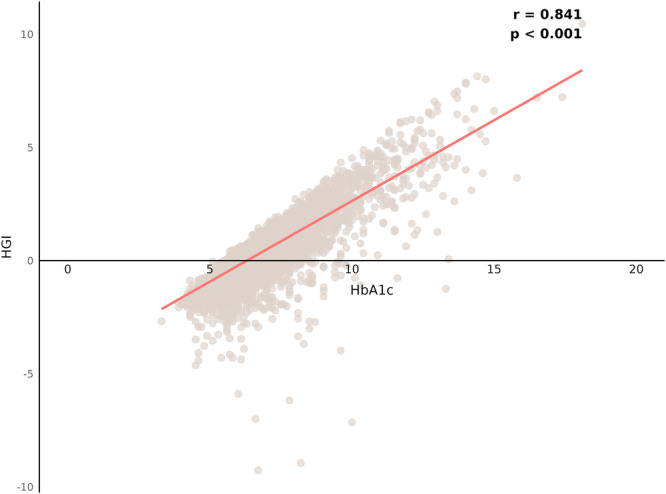
Correlation between the hemoglobin glycation index (HGI) and glycated hemoglobin (HbA1c).

To determine the HGI, a linear regression model was established to evaluate the relationship between FPG and HbA1c using data from all participants included in the study. The predicted HbA1c was then calculated using the derived regression equation: predicted HbA1c = 0.01 × FPG + 4.928. The HGI was subsequently defined as the difference (residual) between the observed and predicted HbA1c values. A positive HGI indicates a higher‐than‐predicted HbA1c level, while a negative HGI indicates a lower‐than‐predicted level. The correlation between HGI and HbA1c was illustrated in Figure 2.

### 2.4 Statistical Analysis

Participants were stratified into quartiles based on HGI values. Continuous variables were summarized as mean ± standard deviation for normally distributed data, or as median with interquartile range for non‐normally distributed data. Categorical variables were presented as frequencies with corresponding percentages. Group comparisons were conducted using Pearson's *χ*² or Fisher's exact tests for categorical data, and Kruskal‐Wallis tests for continuous data, as appropriate. Mortality differences across HGI strata were evaluated using Kaplan‐Meier survival curves with log‐rank tests. The extent of missing data for baseline covariates was assessed and determined to be minimal, with less than 5% missing for all variables (Table S2). A complete case analysis was performed for the multivariable Cox regression models, including only participants with complete data for all covariates in the models. The multivariable Cox proportional hazards models were adjusted for covariates in two sequential tiers: Model 1 included age, sex, BMI, hypertension, diabetes, cardiovascular comorbidities, and organ dysfunction; Model 2 included all variables from Model 1, in addition to laboratory parameters and drug treatment. Restricted cubic splines with four knots were employed to evaluate the nonlinear relationships between HGI and mortality. Subgroup analyses were conducted to examine interactions across various clinical strata, including age, sex, BMI, and comorbidities, utilizing likelihood ratio tests. Mediation models, developed through bootstrapping techniques, were implemented to explore both the direct effects of HGI on diverse clinical outcomes and the indirect effects mediated by RDW and WBC counts. All statistical analyses were performed using R version 4.4.1, with significance determined at a two‐tailed *p*‐value of less than 0.05.

## 3 Results

### 3.1 Baseline Characteristics of Study Subjects

A total of 10,159 patients with atrial fibrillation were included in this study, comprising 6305 males (62.1%, mean age 71.48 ± 11.30 years) and 3854 females (37.9%, mean age 75.04 ± 11.44 years) (Figure 1). Baseline characteristics stratified by HGI quartiles are presented in Table 1. Participants were categorized into four groups according to HGI quartiles: Q1 (HGI ≤ −0.608, *n* = 2,550), Q2 (−0.608 < HGI ≤ −0.208, *n* = 2,557), Q3 (−0.208 < HGI ≤ 0.272, *n* = 2,516), and Q4 (HGI > 0.272, *n* = 2,536).

**Table 1:** Comparison of patients' baseline information.

HGI quartile	Q1 (≤ −0.608)	Q2 (−0.608, −0.208)	Q3 (−0.208, 0.272)	Q4 (> 0.272)	*p*‐value
*N*	2550	2557	2516	2536	
Age (years)	72.81 (64.42; 80.29)	73.73 (65.78; 81.15)	75.36 (67.52; 82.71)	72.61 (65.08; 80.32)	< 0.001
Gender (%)					0.301
Female	973 (38.16%)	945 (36.96%)	990 (39.35%)	946 (37.30%)	
Male	1577 (61.84%)	1612 (63.04%)	1526 (60.65%)	1590 (62.70%)	
Hypertension *n*, (%)	757 (29.69%)	1038 (40.59%)	1206 (47.93%)	1204 (47.48%)	< 0.001
MI *n*, (%)	614 (24.08%)	560 (21.90%)	588 (23.37%)	787 (31.03%)	< 0.001
CHF *n*, (%)	1022 (40.08%)	978 (38.25%)	1069 (42.49%)	1251 (49.33%)	< 0.001
CPD *n*, (%)	555 (21.76%)	629 (24.60%)	741 (29.45%)	733 (28.90%)	< 0.001
Renal disease *n*, (%)	663 (26.00%)	529 (20.69%)	647 (25.72%)	893 (35.21%)	< 0.001
Malignant cancer *n*, (%)	200 (7.84%)	188 (7.35%)	235 (9.34%)	210 (8.28%)	0.065
Diabetes *n*, (%)	574 (22.51%)	465 (18.19%)	750 (29.81%)	2035 (80.24%)	< 0.001
BMI (kg/m^2^)	27.44 (24.23; 31.49)	27.68 (24.39; 31.56)	28.25 (24.70; 32.45)	30.31 (26.24; 35.11)	< 0.001
Hemoglobin, g/L	9.60 (8.10; 11.20)	9.80 (8.40; 11.40)	9.70 (8.30; 11.20)	9.70 (8.30; 11.30)	0.004
RDW	13.80 (13.10; 15.00)	13.70 (13.00; 14.60)	13.80 (13.20; 14.70)	14.00 (13.30; 15.00)	< 0.001
Platelets, 10^9^/L	146.00 (106.00; 200.00)	148.00 (110.00; 203.00)	149.00 (113.00; 199.50)	164.00 (123.00; 220.00)	< 0.001
WBC, 10^9^/L	13.60 (9.90; 18.20)	13.50 (10.00; 17.80)	13.20 (9.60; 17.80)	13.30 (9.60; 17.80)	0.283
Blood creatinine, mg/dL	1.10 (0.80; 1.60)	1.00 (0.80; 1.40)	1.10 (0.80;1.40)	1.20 (0.90; 1.80)	< 0.001
INR	1.40 (1.20; 1.70)	1.40 (1.20; 1.60)	1.40 (1.20; 1.70)	1.40 (1.20; 1.70)	0.132
APSIII	41.00 (30.00; 54.00)	37.00 (28.00; 49.00)	39.00 (30.00; 50.00)	43.00 (32.00; 56.00)	< 0.001
SOFA	5.00 (3.00; 7.00)	4.00 (2.00; 6.00)	4.00 (3.00; 7.00)	4.00 (3.00; 7.00)	< 0.001
Insulin *n*, (%)	2007 (78.71%)	1988 (77.75%)	1974 (78.46%)	2352 (92.74%)	< 0.001
Vasoactive drug *n*, (%)	531 (20.82%)	483 (18.89%)	513 (20.39%)	545 (21.49%)	< 0.001
β‐Blocker *n*, (%)	1749 (68.59%)	1695 (66.29%)	1712 (68.04%)	1763 (69.52%)	< 0.001
30‐day mortality (%)	271 (10.63)	186 (7.27)	238 (9.46)	232 (9.15)	< 0.001
90‐day mortality (%)	395 (15.49)	282 (11.03)	343 (13.63)	344 (13.56)	< 0.001
365‐day mortality (%)	584 (22.90)	440 (17.21)	515 (20.47)	559 (22.04)	< 0.001
Length of ICU stay, day	2.68 (1.51; 5.13)	2.29 (1.39; 4.31)	2.44 (1.47; 4.36)	2.36 (1.45; 4.29)	< 0.001

Abbreviations: APSII, acute physiology score II; BMI, body mass index; CHF, congestive heart failure; CPD, chronic pulmonary disease; ICU, intensive care unit; INR, international normalized ratio; MI, myocardial infarction; SOFA, sequential organ failure assessment; WBC, white blood cell.

Compared with patients in the lower HGI quartiles, those in the highest quartile (Q4) exhibited significantly higher BMI, and a greater prevalence of myocardial infarction, congestive heart failure, chronic pulmonary disease, and renal disease. Additionally, they had elevated platelet counts, higher serum creatinine levels, and a greater proportion of insulin users.

### 3.2 Survival Analysis

**Figure 3:**
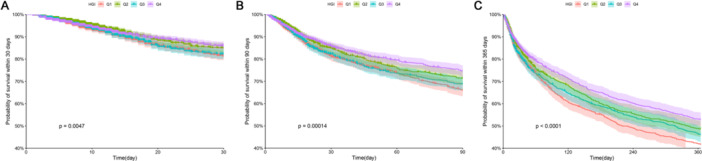
Kaplan‐Meier survival curves illustrating all‐cause mortality across HGI quartiles. (A) Comparison of 30‐day mortality between quartile groups. (B) Comparison of 90‐day mortality between quartile groups. (C) Comparison of 365‐day mortality between quartile groups.

Of the 10,159 patients included in the study, 927 (9.12%), 1364 (13.43%), and 2098 (20.65%) succumbed within 30, 90, and 365 days of follow‐up, respectively. Kaplan‐Meier survival analysis was conducted to evaluate differences in mortality rates among patients stratified into quartiles based on their HGI. Notably, patients in the HGI Q1 group demonstrated significantly elevated all‐cause mortality rates at 30, 90, and 365 days compared to the other quartile groups. Furthermore, statistically significant differences in mortality rates were observed across the four quartiles, as indicated by log‐rank *p*‐values of 0.0047, 0.00014, and < 0.0001, respectively. The detailed findings of the analysis are presented in Figure 3.

### 3.3 Correlation of the HGI With Outcome Events

In the analysis of patient baseline characteristics, the Q2 group (−0.608 < HGI ≤ −0.208) demonstrated the lowest mortality rate among all groups and was designated as the reference group in Cox proportional hazards models assessing the association between HGI and all‐cause mortality over 30, 90, and 365 day, as shown in Table 2. When treating HGI as a continuous variable, for 30‐day mortality, higher HGI values were associated with a statistically significant reduction in mortality risk in the unadjusted model (hazard ratio (HR) = 0.91 (0.86–0.97), *p* = 0.002). However, this association was attenuated to borderline significance after full adjustment (HR = 0.92 (0.85–1.00), *p* = 0.058). Similar trends were observed for 90‐day (HR = 0.89 (0.83–0.95), *p* = 0.001) and 365‐day mortality (HR = 0.89 (0.84–0.94), *p* < 0.001) in fully adjusted models. Quartile analyses indicated notable threshold effects: compared with Q2, the lowest HGI quartile (Q1) exhibited significantly higher mortality risks of 46% (HR = 1.46 (1.13–1.89), *p* = 0.004), 42% (HR = 1.42 (1.15–1.76), *p* = 0.001), and 26% (HR = 1.26 (1.06–1.50), *p* = 0.007) at 30, 90, and 365 days, respectively. In contrast, the highest quartile (Q4) did not exhibit significant differences from Q2 (all *p* > 0.4) (Table 2). These findings suggest that individuals in the lowest HGI quartile face substantially elevated risks.

**Table 2:** Multivariate Cox regression analyses for 30‐day, 90‐day, and 365‐day mortality.

Variable	Non‐adjusted		Model 1		Model 2	
	HR (95%CI)	*p*‐value	HR (95%CI)	*p*‐value	HR (95%CI)	*p*‐value
**All‐cause mortality within 30 days**						
HGI continuous	0.91 (0.86–0.97)	0.002	0.91 (0.83–1.00)	0.04	0.92 (0.85–1.00)	0.058
HGI group Q1	1.32 (1.10–1.60)	0.003	1.53 (1.19–1.96)	< 0.001	1.46 (1.13–1.89)	0.004
HGI group Q2	Ref		Ref		Ref	
HGI group Q3	1.24 (1.03–1.51)	0.026	1.34 (1.03–1.74)	0.028	1.42 (1.08–1.85)	0.011
HGI group Q4	1.04 (0.86–1.26)	0.709	1.11 (0.83–1.49)	0.483	1.12 (0.84–1.51)	0.44
**All‐cause mortality within 90 days**						
HGI continuous	0.89 (0.84–0.93)	< 0.001	0.89 (0.83–0.96)	0.001	0.89 (0.83–0.95)	0.001
HGI group Q1	1.28 (1.10–1.49)	0.002	1.44 (1.17–1.77)	< 0.001	1.42 (1.15–1.76)	0.001
HGI group Q2	Ref		Ref		Ref	
HGI group Q3	1.18 (1.00–1.38)	0.044	1.25 (1.01–1.55)	0.043	1.32 (1.05–1.66)	0.015
HGI group Q4	0.95 (0.81–1.11)	0.515	1.04 (0.82–1.31)	0.774	1.04 (0.81–1.33)	0.767
**All‐cause mortality within 365 days**						
HGI continuous	0.89 (0.86–0.93)	< 0.001	0.89 (0.84–0.94)	< 0.001	0.89 (0.84–0.94)	< 0.001
HGI group Q1	1.25 (1.10–1.41)	< 0.001	1.29 (1.10–1.52)	0.002	1.26 (1.06–1.50)	0.007
HGI group Q2	Ref		Ref		Ref	
HGI group Q3	1.12 (0.99–1.28)	0.073	1.11 (0.94–1.33)	0.224	1.16 (0.97–1.39)	0.111
HGI group Q4	0.93 (0.82–1.05)	0.233	0.94 (0.78–1.13)	0.509	0.95 (0.78–1.15)	0.599

*Note:* HGI: Q1 (HGI ≤ −0.608), Q2 (−0.608 < HGI ≤ −0.208), Q3 (−0.208 < HGI ≤ 0.272), and Q4 (HGI > 0.272). CI, confidential interval; HR, hazard ratio.

Model 1: adjusted for age, sex, BMI, hypertension, diabetes, myocardial infarction, congestive heart failure, malignant cancer, chronic pulmonary disease, and renal disease.

Model 2: adjusted for age, sex, BMI, hypertension, diabetes, myocardial infarction, congestive heart failure, malignant cancer, chronic pulmonary disease, renal disease, white blood cell, red blood cell distribution width, hemoglobin, international normalized ratio, creatinine, calcium, potassium, sodium, insulin, vasoactive drug, and β‐blocker.

**Figure 4:**
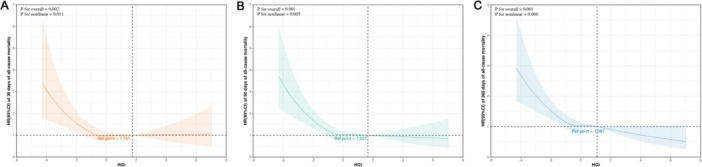
Associations between HGI and all‐cause mortality in critically ill patients with atrial fibrillation. (A) 30‐day mortality; (B) 90‐day mortality; (C) 365‐day mortality. Vertical dashed lines represent inflection points, dark gray lines denote fully adjusted risk ratios, shaded areas indicate 95% confidence intervals and horizontal dashed lines represent the hazard ratio of 1.

The restricted cubic spline model was used to examine the non‐linear association between HGI and all‐cause mortality. For 30‐day mortality, a significant non‐linear association was identified (*p* for overall = 0.002; *p* for non‐linear = 0.011). The risk (HR) increased as HGI levels decreased, while it remained flat and non‐significant above the reference point of 1.741 (Figure 4A). A similar, more pronounced non‐linear trend was observed for 90‐day mortality (*p* for overall < 0.001; *p* for non‐linear = 0.005) (Figure 4B). For 365‐day mortality, the overall pattern was similar, but the non‐linear trend was less pronounced and did not reach statistical significance (*p* for non‐linear = 0.060), suggesting reduced statistical power at this longer time point (Figure 4C). Collectively, these findings indicate that the association between HGI and mortality is non‐linear and inverse, with the risk being primarily driven by the adverse effects of lower HGI values.

### 3.4 Subgroup Analysis

**Figure 5:**
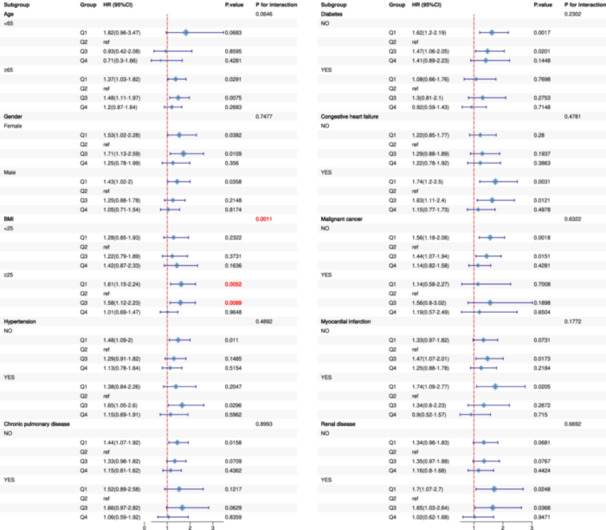
Subgroup analysis of 30‐day mortality by demographic and clinical characteristics.

**Figure 6:**
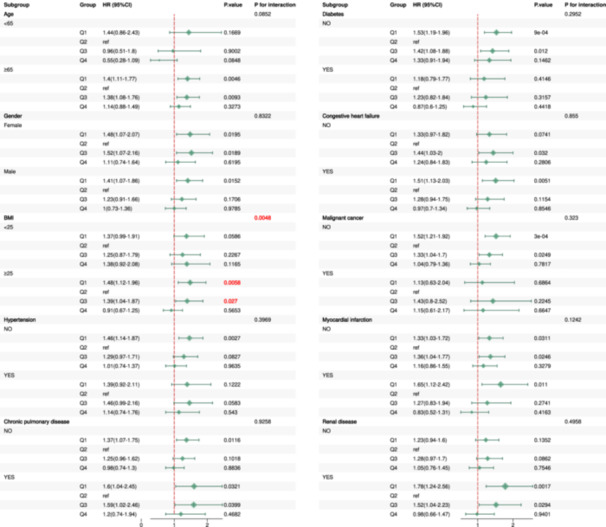
Subgroup analysis of 90‐day mortality by demographic and clinical characteristics.

**Figure 7:**
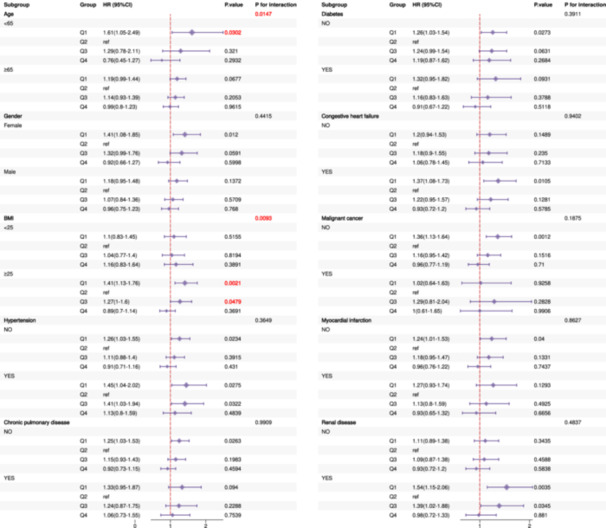
Subgroup analysis of 365‐day mortality by demographic and clinical characteristics.

The stratified analyses revealed that the prognostic impact of HGI varied across patient subgroups, with BMI emerging as a significant and consistent effect modifier. Patients with BMI ≥ 25 exhibited strong Q1‐associated risks across all timeframes (HRs ranging from 1.41 to 1.61, all *p*< 0.01), and the tests for interaction were highly significant for 30‐day (*p* for interaction = 0.001), 90‐day (*p* for interaction = 0.005), and 365‐day mortality (*p* for interaction = 0.009). In the subgroup of patients aged ≥ 65 years, a significant interaction was also detected, but only for 365‐day mortality (*p* for interaction = 0.015). While elevated risks were observed within other specific subgroups (e.g., those without certain comorbidities or with organ dysfunction, as detailed in Figures 5, 6, 7), the formal tests for interaction were not statistically significant for these characteristics. Collectively, these findings highlight that the prognostic significance of HGI is most strongly and consistently modified by BMI. A significant interaction was also noted for age, specifically for 365‐day mortality. These results underscore the importance of considering patient characteristics, particularly BMI, in HGI‐based risk stratification and emphasize the need for further investigation into the underlying mechanisms.

### 3.5 Mediation Analysis

First, we assessed the prognostic value of the potential mediators. As shown in Table S3, higher RDW was significantly associated with increased mortality at all time points, even after multivariable adjustment (all *p* < 0.05). We then performed causal mediation analyses to test whether RDW or WBC mediated the association between HGI and mortality. WBC showed no significant mediating effect in any of the analyses (Tables S4–S6).

**Figure 8:**
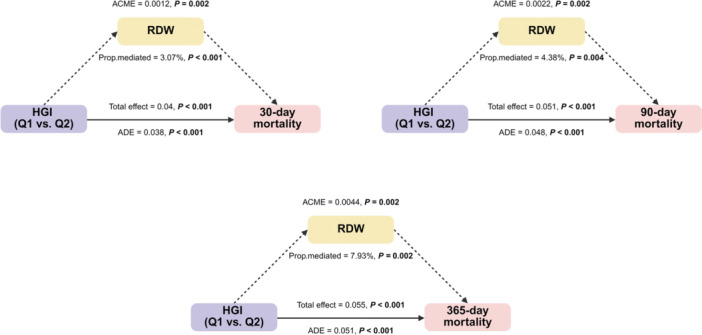
Path diagram of the mediation analysis of RBC on the relationship between the HGI and 30‐day, 90‐day, and 365‐day mortality, respectively. ACME (average causal mediation effect), ADE (average direct effect).

In contrast, RDW was identified as a significant, albeit partial, mediator of the risk associated with the lowest HGI quartile (Q1 vs. Q2). Specifically, RDW mediated 3.07% (*p *< 0.001), 4.38% (*p *= 0.004), and 7.93% (*p *= 0.002) of the total effect of low HGI on 30‐day, 90‐day, and 365‐day mortality, respectively (Figure 8). This mediating effect was specific to the Q1 group, as no significant mediation by RDW was observed for the Q3 or Q4 HGI quartiles (Tables S4–S6).

## 4 Discussion

In this study, we sought to assess the relationship between the HGI and the prognosis of critically ill patients with atrial fibrillation, utilizing data from the MIMIC‐IV database. Our extensive cohort analysis, encompassing 10,159 critically ill patients with atrial fibrillation, identified a non‐linear association between HGI and all‐cause mortality. Notably, patients in the lowest HGI quartile (Q1) exhibited a 26%–46% increased risk of mortality over a period ranging from 30 to 365 days.

Our findings contrast with previous research on non‐alcoholic fatty liver disease and diabetes, where elevated hemoglobin glycation index (HGI) has been consistently linked to adverse outcomes [15, 16, 17, 18]. In our study, we found that a low HGI was significantly associated with both short‐ and long‐term mortality, while a high HGI did not present a significant risk. Furthermore, when HGI was analyzed as a continuous variable, higher HGI values correlated with a statistically significant decrease in 90‐ and 365‐day mortality in the unadjusted model. This discrepancy is likely attributable to the distinct pathophysiological mechanisms specific to atrial fibrillation (AF). A low HGI may indicate impaired erythrocyte turnover, malnutrition, or frailty, factors that are strongly associated with poor prognosis in critically ill populations [19, 20]. Individuals with low HGI exhibit a mismatch between HbA1c levels and actual blood glucose levels, potentially leading to under‐treatment with glucose‐lowering therapies and an elevated risk for diabetes‐related complications [21]. Consistent with our findings, prior studies using the MIMIC‐IV database in patients with coronary artery disease, heart failure, and stroke have similarly demonstrated that low HGI is associated with increased mortality [9, 22]. The absence of increased risk in high HGI, despite a greater prevalence of comorbidities, may be attributable to unmeasured confounders, such as intensified clinical monitoring, or protective mechanisms, such as better diabetes management with oral hypoglycemic agents.

The observed threshold effect (Q1 vs. Q2–Q4) aligns with existing evidence suggesting that extreme deviations in glycation markers influence cardiovascular risk [21]. In the context of AF, the link between low HGI and adverse outcomes may be explained by a cascade of interconnected pathophysiological mechanisms rooted in erythrocyte dysfunction. Specifically, regarding erythrocyte dynamics, a consistently low HGI often serves as a surrogate marker for accelerated RBC destruction and shortened lifespan [23]. The chronic pro‐inflammatory and high‐oxidative‐stress environment inherent to AF can directly damage RBCs [24]. This oxidative damage targets the RBC membrane, leading to lipid peroxidation and protein cross‐linking, which in turn severely impairs erythrocyte deformability [25]. These resulting rigid, less pliable erythrocytes struggle to traverse the microcirculation, particularly the coronary capillaries. This can lead to microvascular sludging and obstruction, thereby compromising oxygen delivery to the myocardium even at baseline [26]. During tachyarrhythmic episodes in AF, when myocardial oxygen demand drastically increases, this pre‐existing microvascular insufficiency creates a critical supply‐demand mismatch, profoundly exacerbating myocardial ischemia. Furthermore, damaged and dysfunctional RBCs are cleared more rapidly by the reticuloendothelial system, which shortens their circulating lifespan and thus provides a mechanistic basis for the observed low HGI [27].

Supporting this proposed mechanism, our causal mediation analysis provides direct evidence for this pathway. We found that RDW, a well‐established indicator of erythrocyte anisocytosis and impaired RBC homeostasis, significantly mediated the association between low HGI and increased mortality at all follow‐up periods. Although the mediated proportion was modest (3%–8%), this finding empirically links the prognostic risk of low HGI to the health status of red blood cells. Furthermore, our analysis demonstrated that WBC count, a general marker of systemic inflammation, did not act as a significant mediator. This suggests that the risk conferred by low HGI may be more specific to pathways involving erythropoietic stress and rheological dysfunction, represented by RDW, rather than a non‐specific systemic inflammatory response.

We observed a temporal trend in the association between HGI and mortality, with HRs decreasing from 1.46 at 30 days to 1.26 at 365 days. This trend suggests that HGI may have greater prognostic value during the acute phase of critical illness. Several factors may account for this observation. Firstly, low HGI is driven by pathophysiological mechanisms characterized by increased erythrocyte turnover, hemolysis, and acute oxidative stress; these often signal severe acute physiological disturbances such as sepsis and acute kidney injury that carry a high risk of early mortality. Patients who survive beyond this critical period are likely to have stabilized, and their long‐term outcomes may be more influenced by chronic comorbidities rather than the acute conditions reflected by HGI. Therefore, HGI serves as a particularly effective marker for early risk stratification in the ICU, enabling clinicians to identify patients who require immediate and intensive monitoring.

Subgroup analyses further underscored the prognostic relevance of HGI in specific populations. For example, Q1‐associated risks were amplified in obese patients (BMI ≥ 25), potentially reflecting the exacerbation of HGI's metabolic effects by insulin resistance [28, 29]. This observed synergistic effect may be rooted in a vicious cycle of inflammation and oxidative stress, common to both conditions. Obesity and insulin resistance are characterized by a chronic, low‐grade pro‐inflammatory state, driven by adipokines released from adipose tissue [30]. This systemic inflammation can independently shorten RBC lifespan, contributing to a lower HGI [31]. Concurrently, as previously discussed, a low HGI itself signifies underlying RBC fragility and rheological impairment. Therefore, in obese patients, the pre‐existing inflammatory milieu may both cause and amplify the adverse effects of low HGI, creating a compounded rheological and ischemic burden that could explain the markedly worsened outcomes.

These findings also have clinical implications. First, low HGI may serve as an indicator for identifying high‐risk AF patients who could benefit from closer clinical monitoring, tailored glycemic management, or nutritional interventions. Second, the lack of excess risk in the high‐HGI subgroup, despite a higher comorbidity burden, suggests that current glycemic management strategies, such as intensified monitoring or the use of oral hypoglycemic agents, may mitigate risks in these patients. However, further validation in ambulatory and non‐ICU cohorts is warranted to generalize these findings and explore their applicability in broader populations.

### 4.1 Limitations

This study has several limitations. First, the potential for residual confounding due to unmeasured variables, such as erythrocyte lifespan, dietary patterns, and pharmacologic interventions, cannot be excluded. Second, as our analysis was based on the MIMIC‐IV database, which focuses exclusively on ICU patients, the findings may not be generalizable to ambulatory AF populations. Third, HGI was calculated using single‐timepoint fasting glucose measurements, which may not adequately capture glycemic variability in the context of critical illness. Future studies should incorporate dynamic glucose monitoring and additional biomarkers, such as erythrocyte turnover or oxidative stress markers, to better contextualize the role of HGI in critical care.

## 5 Conclusion

In conclusion, this study identifies a significant, non‐linear inverse association between HGI and mortality in patients with atrial fibrillation, where the risk is predominantly driven by values in the lowest quartile. The prognostic significance of HGI was particularly pronounced in patients with elevated BMI. Furthermore, our mediation analysis revealed that RDW, a marker of red blood cell dysfunction, partially mediated this risk, particularly in the lowest HGI quartile, suggesting a tangible link between HGI and erythropoietic stress. Future research should focus on validating these findings in non‐ICU cohorts, exploring the mechanisms underlying low HGI, and integrating HGI into clinical workflows to improve risk stratification and therapeutic decision‐making.

## Author Contributions

**Xianglin Long:** methodology, software, writing–original draft, investigation, resources, writing–review and editing. **Wei Chen:** conceptualization, methodology, formal analysis, data curation, supervision, writing–review and editing. All authors contributed to the article and approved the submitted version.

## Ethics Statement

This research was conducted in compliance with the Helsinki Declaration's guidelines. The original data collection for the MIMIC‐IV database was approved by the Institutional Review Boards of Beth Israel Deaconess Medical Center (Approval No. 2001‐P‐001699/14) and the Massachusetts Institute of Technology (Approval No. 0403000206).

## Consent

As the database contains completely de‐identified clinical data, the requirement for individual informed consent was waived by the respective IRBs, obviating the need for further ethical approval for this specific retrospective study.

## Conflicts of Interest

The authors declare no conflicts of interest.

## Supporting information

Supporting File

## Data Availability

The datasets generated and analyzed in this study are available at the MIMIC‐IV website: https://physionet.org/content/mimiciv/3.1/.
