# Supplementary material for: Association Between Hemoglobin Glycation Index and Mortality in Critically Ill Patients With Atrial Fibrillation: A Retrospective Cohort Study
Source: Clin Cardiol. 2026 Apr 22;49(4):e70321. doi: 10.1002/clc.70321 (PMC13100645; doi:10.1002/clc.70321)
Supplement: Supplementary file 1 — Supporting File [file CLC-49-e70321-s001.docx]

Supplementary Information

**Association between hemoglobin glycation index and mortality in critically ill patients with atrial fibrillation: a retrospective cohort study**

**Supplementary Table S1.**

**The meaning of ICD-9 and ICD-10 codes for diseases**

| Variables | ICD codes | Meaning of diagnostic code |
| --- | --- | --- |
| atrial fibrillation | 42731 | Atrial fibrillation |
|  | I480 | Paroxysmal atrial fibrillation |
|  | I481 | Persistent atrial fibrillation |
|  | I4811 | Longstanding persistent atrial fibrillation |
|  | I4819 | Other persistent atrial fibrillation |
|  | I482 | Chronic atrial fibrillation |
|  | I4820 | Chronic atrial fibrillation, unspecified |
|  | I4821 | Permanent atrial fibrillation |
|  | I4891 | Unspecified atrial fibrillation |

Abbreviation: ICD: international classification of diseases

**Supplementary Table S2.**

**Missing number of variables**

| **Variables** | **Miss.freq** | **Miss.percentage** |
| --- | --- | --- |
| BMI | 85 | 0.0084 |
| WBC | 37 | 0.0036 |
| RDW | 19 | 0.0019 |
| Hemoglobin | 35 | 0.0034 |
| Platelets | 38 | 0.0037 |
| Calcium | 307 | 0.0302 |
| Blood creatinine | 25 | 0.0025 |
| Sodium | 36 | 0.0035 |
| Potassium | 50 | 0.0049 |
| INR | 432 | 0.0425 |
| APSIII | 0 | 0 |
| SOFA | 0 | 0 |
| Hypertension | 0 | 0 |
| MI | 0 | 0 |
| CHF | 0 | 0 |
| CPD | 0 | 0 |
| Renal disease | 0 | 0 |
| Malignant cancer | 0 | 0 |
| Diabetes | 0 | 0 |
| Insulin | 0 | 0 |
| Vasoactive drug | 0 | 0 |
| β-blocker | 0 | 0 |

Abbreviations: BMI, Body mass index; WBC, White blood cell; RDW, Red blood cell distribution width; INR, International normalized ratio; APSIII, Acute physiology score III; SOFA, Sequential Organ Failure Assessment; MI, Myocardial infarction; CHF, Congestive Heart failure; CPD, Chronic pulmonary disease.

**Supplementary Table S3.**

**The associations between mediating variables and 30-day, 90-day, and 365-day mortality**

| Variables | Outcome | Non-adjusted | | Adjusted model | |
| --- | --- | --- | --- | --- | --- |
|  |  | HR (95% CI) | P-value | HR (95% CI) | P-value |
| RDW | 30-day mortality | 2.59 (1.31-5.12) | 0.006 | 2.18 (1.07-4.46) | 0.032 |
|  | 90-day mortality | 2.56 (1.45-4.50) | 0.001 | 2.17 (1.21-3.89) | 0.009 |
|  | 365-day mortality | 3.15 (1.99-4.97) | <0.001 | 2.71 (1.69-4.34) | <0.001 |
| WBC | 30-day mortality | 0.98 (0.83-1.17) | 0.863 | 1.02 (0.87-1.21) | 0.799 |
|  | 90-day mortality | 1.04 (0.90-1.21) | 0.554 | 1.07 (0.93-1.22) | 0.359 |
|  | 365-day mortality | 1.03 (0.92-1.16) | 0.634 | 1.02 (0.91-1.14) | 0.715 |

HR: Hazard Ratio, CI: Confidence Interval

Adjusted model: Adjusted for age, sex, BMI, hypertension, diabetes, myocardial infarction, congestive heart failure, malignant cancer, chronic pulmonary disease, renal disease, hemoglobin, international normalized ratio, creatinine, calcium, potassium, sodium, insulin, vasoactive drug, and β-blocker, and adjusted mutually for the mediating variables.

RDW and WBC were log-transformed in the models.

**Supplementary Table S4.**

**Mediating effects of RDW and WBC on the association between HGI and 30-day mortality**

| Variables | Total effect (95%CI) | P | ACME (95%CI) | P | ADE (95%CI) | P | Prop. mediated (95%CI) | P |
| --- | --- | --- | --- | --- | --- | --- | --- | --- |
| RDW (Q1 vs. Q2) | 0.040 (0.023 ~ 0.058) | <0.001 | 0.0012 (0.0004 ~ 0.0023) | <0.001 | 0.038 (0.021 ~ 0.056) | <0.001 | 3.07% (0.90% ~ 7.20%) | <0.001 |
| RDW (Q3 vs. Q2) | 0.014 (-0.002 ~ 0.031) | 0.082 | 0.0001 (-0.0006 ~ 0.0009) | 0.816 | 0.014 (-0.002 ~ 0.031) | 0.078 | 0.51% (-10.68% ~ 13.71%) | 0.822 |
| RDW (Q4 vs. Q2) | 0.007 (-0.012 ~ 0.025) | 0.45 | -0.0001 (-0.0010 ~ 0.0008) | 0.792 | 0.007 (-0.012 ~ 0.025) | 0.452 | -1.98% (-53.59% ~ 31.97%) | 0.942 |
| WBC (Q1 vs. Q2) | 0.038 (0.019 ~ 0.057) | <0.001 | -0.00004 (-0.0003 ~ 0.0002) | 0.878 | 0.038 (0.019 ~ 0.057) | <0.001 | -0.10% (-0.99% ~ 0.74%) | 0.878 |
| WBC (Q3 vs. Q2) | 0.014 (-0.003 ~ 0.031) | 0.122 | -0.00005 (-0.0004 ~ 0.0002) | 0.738 | 0.014 (-0.003 ~ 0.031) | 0.122 | -0.39% (-6.05% ~ 6.37%) | 0.756 |
| WBC (Q4 vs. Q2) | 0.007 (-0.012 ~ 0.028) | 0.444 | 0.00002 (-0.0002 ~ 0.0004) | 0.818 | 0.007 (-0.012 ~ 0.028) | 0.45 | 0.30% (-11.40% ~ 12.53%) | 0.87 |

All models were adjusted. Mediation analysis performed using log-transformed RDW and WBC as the mediator variable.

**Supplementary Table S5.**

**Mediating effects of RDW and WBC on the association between HGI and 90-day mortality**

| Variables | Total effect (95%CI) | P | ACME (95%CI) | P | ADE (95%CI) | P | Prop. mediated (95%CI) | P |
| --- | --- | --- | --- | --- | --- | --- | --- | --- |
| RDW (Q1 vs. Q2) | 0.051 (0.028 ~ 0.074) | <0.001 | 0.0022 (0.0009 ~ 0.0039) | 0.004 | 0.048 (0.025 ~ 0.070) | <0.001 | 4.38% (1.54% ~ 10.60%) | 0.004 |
| RDW (Q3 vs. Q2) | 0.009 (-0.010 ~ 0.028) | 0.364 | 0.0001 (-0.0010 ~ 0.0015) | 0.852 | 0.009 (-0.011 ~ 0.028) | 0.370 | 1.44% (-71.90% ~ 60.45%) | 0.952 |
| RDW (Q4 vs. Q2) | 0.003 (-0.017 ~ 0.026) | 0.702 | -0.0002 (-0.0018 ~ 0.0013) | 0.748 | 0.004 (-0.017 ~ 0.026) | 0.704 | -7.07% (-69.53% ~ 97.76%) | 0.938 |
| WBC (Q1 vs. Q2) | 0.048 (0.025 ~ 0.070) | <0.001 | -0.0001 (-0.0005 ~ 0.0003) | 0.770 | 0.048 (0.025 ~ 0.070) | <0.001 | -0.15% (-1.14% ~ 0.53%) | 0.770 |
| WBC (Q3 vs. Q2) | 0.009 (-0.011 ~ 0.029) | 0.362 | -0.0001 (-0.0026 ~ 0.0002) | 0.592 | 0.009 (-0.011 ~ 0.029) | 0.358 | -1.17% (-20.78% ~ 9.40%) | 0.722 |
| WBC (Q4 vs. Q2) | 0.004 (-0.017 ~ 0.026) | 0.690 | 0.0000 (-0.0003 ~ 0.0005) | 0.802 | 0.004 (-0.017 ~ 0.026) | 0.700 | -1.05% (-14.09% ~ 17.02%) | 0.908 |

All models were adjusted. Mediation analysis performed using log-transformed RDW and WBC as the mediator variable.

**Supplementary Table S6.**

**Mediating effects of RDW and WBC on the association between HGI and 365-day mortality**

| Variables | Total effect (95%CI) | P | ACME (95%CI) | P | ADE (95%CI) | P | Prop. mediated (95%CI) | P |
| --- | --- | --- | --- | --- | --- | --- | --- | --- |
| RDW (Q1 vs. Q2) | 0.055 (0.029 ~ 0.080) | <0.001 | 0.0044 (0.0016 ~ 0.0077) | 0.002 | 0.051 (0.025 ~ 0.074) | <0.001 | 7.93% (2.75% ~ 17.15%) | 0.002 |
| RDW (Q3 vs. Q2) | -0.006 (-0.028 ~ 0.020) | 0.686 | 0.0003 (-0.0020 ~ 0.0026) | 0.828 | -0.006 (-0.028 ~ 0.019) | 0.674 | -4.64% (-118.26% ~ 145.63%) | 0.970 |
| RDW (Q4 vs. Q2) | 0.000 (-0.025 ~ 0.027) | 0.932 | -0.0005 (-0.0037 ~ 0.0023) | 0.684 | 0.000 (-0.024 ~ 0.028) | 0.902 | -28.50% (-192.74% ~ 139.42%) | 1.000 |
| WBC (Q1 vs. Q2) | 0.050 (0.025 ~ 0.074) | <0.001 | -0.0003 (-0.0010 ~ 0.0004) | 0.414 | 0.050 (0.025 ~ 0.074) | <0.001 | -0.52% (-2.48% ~ 0.91%) | 0.414 |
| WBC (Q3 vs. Q2) | -0.006 (-0.028 ~ 0.019) | 0.688 | -0.0004 (-0.0013 ~ 0.0003) | 0.266 | -0.005 (-0.027 ~ 0.019) | 0.706 | 7.04% (-59.00% ~ 54.92%) | 0.754 |
| WBC (Q4 vs. Q2) | 0.002 (-0.024 ~ 0.028) | 0.898 | 0.0001 (-0.0007 ~ 0.0012) | 0.726 | 0.002 (-0.024 ~ 0.028) | 0.904 | -6.22% (-36.14% ~ 29.42%) | 0.932 |

All models were adjusted. Mediation analysis performed using log-transformed RDW and WBC as the mediator variable.
